# Dynamics of nucleic acid mobility

**DOI:** 10.1093/genetics/iyad132

**Published:** 2023-07-26

**Authors:** Shailja Singh, Xinyi Hu, Christina Dixelius

**Affiliations:** Department of Plant Biology, Uppsala BioCenter, Linnéan Center for Plant Biology, Swedish University of Agricultural Sciences, P.O. Box 7080, Uppsala, SE-75007, Sweden; Department of Plant Biology, Uppsala BioCenter, Linnéan Center for Plant Biology, Swedish University of Agricultural Sciences, P.O. Box 7080, Uppsala, SE-75007, Sweden; Department of Plant Biology, Uppsala BioCenter, Linnéan Center for Plant Biology, Swedish University of Agricultural Sciences, P.O. Box 7080, Uppsala, SE-75007, Sweden

**Keywords:** vesicles, endoplasmic reticulum, membrane-less organelles

## Abstract

Advances in sequencing technologies and bioinformatic analyses are accelerating the quantity and quality of data from all domains of life. This rich resource has the potential to reveal a number of important incidences with respect to possible exchange of nucleic acids. Ancient events have impacted species evolution and adaptation to new ecological niches. However, we still lack a full picture of processes ongoing within and between somatic cells, gametes, and different organisms. We propose that events linked to acceptance of alien nucleic acids grossly could be divided into 2 main routes in plants: one, when plants are exposed to extreme challenges and, the second level, a more everyday or season-related stress incited by biotic or abiotic factors. Here, many events seem to comprise somatic cells. Are the transport and acceptance processes of alien sequences random or are there specific regulatory systems not yet fully understood? Following entrance into a new cell, a number of intracellular processes leading to chromosomal integration and function are required. Modification of nucleic acids and possibly exchange of sequences within a cell may also occur. Such fine-tune events are most likely very common. There are multiple questions that we will discuss concerning different types of vesicles and their roles in nucleic acid transport and possible intracellular sequence exchange between species.

## Introduction

The question of a continuous flow of genes between prokaryotes and between prokaryotes and eukaryotes beyond endosymbiotic-related events involving mitochondria and plastids has received much attention (Keeling and Palmer 2008; Ku *et al.* 2015). Prokaryote to prokaryote exchange is common, resulting in dynamic genomic features through gains and losses of genes (Ochman *et al.* 2000; Lapierre and Gogarten 2009; Lobkovsky *et al.* 2013; Vernikos *et al.* 2015). In an extensive search for recent horizontal gene transfer (HGT) events from prokaryotes to eukaryotes, it was concluded that eukaryotes do not acquire genes continuously from distantly related organisms, as seen between prokaryotes (Ku and Martin 2016). Is that true? What happens when eukaryotic cells and organisms are exposed to challenges from external cues? How could posttranscriptional gene regulation and posttranslational modifications possibly alter the genetic legacy in a long-term perspective? These are to large degrees open fundamental questions but are important to understand to leverage solutions to emergent problems, not least in a climate change context.

Land plants (embryophytes) have evolved from freshwater communities, particularly from streptophytes (de Vries and Archibald 2018). Numerous events have impacted the rich diversity of plant species known today, including the acquisition of gene families from other taxa such as fungi and bacteria (Ma *et al.* 2022). These gene transfers have mainly occurred during dramatic and challenging time periods of plant evolution: the early evolution of streptophytes and the origin of land plants. Various stress factors seem to be of outstanding importance in situations where we today have started to identify genetic traces from alien taxa. This observation could be biased; it may just be easier to detect new genetic influx or exchanges in such cases. Further, the sequence information available in the databases are not unbiased. So far, there is a preponderance of species of human interests, aggravating detection of influx or exchange as a result of daily environmental exposures. Numerous questions remain to be elucidated such as the following: what are the specific conditions, if any, that promote transfer of nucleic acid between organisms? And if so, are there specific thresholds? Is tight physical contact required between the donor and the recipient partner to allow any type of genetic exchange? There is great many of such examples involving pathogens or parasitic organisms that form specific infection structures in plants. However, nonpathogenic endophytes have over time delivered genes to both prokaryotes and eukaryotes including plants (Tiwari and Bae 2020). One important example from the latter category is the acquired glutathione S-transferase gene by wheatgrass used as a resistance resource in wheat breeding to Fusarium head blight (Wang *et al.* 2020). Whether the cell walls had been degraded or somehow damaged to facilitate this gene transfer is not known. Why is the alien nucleic acid not recognized and degraded by nucleases in the new host cell? If entering the nucleus, what about sequence-dependent recognition signatures, choice of integration site, and proper gene function? Is it a constant external flow of nucleic acid and only that the rare successful events are noticed? Last but not least, isolated events progressing to population levels require gene transmission to offspring and evolutionary advantages of new traits. In the following sections, we will discuss organelles and cellular structures that could possibly aid or support influx and efflux of nucleic acids in plant cells particularly under abiotic or biotic stress situations.

## Transport of nucleic acids in membrane-enclosed vesicles

Eukaryotic cells contain many different compartments in the cytosol and nucleus. They can grossly be divided into membrane-enclosed and membrane-less organelles. First, we will discuss the membrane-enclosed vesicles. The vesicle membrane composition varies between species as well as the vesicle content and cargo target (Gould *et al.* 2016; Tashiro *et al.* 2019). Vesicles are produced continuously in all organisms but are most abundantly produced under stressful conditions. Extracellular vesicles (EVs) are of importance for both gram-negative and gram-positive pathogenic bacteria in their animal host cell interactions, a research field where most observations have been generated to date (Gill *et al.* 2019; Toyofuku *et al.* 2019; Briaud and Carroll 2020). In gram-negative bacteria, EVs are pinched off from the outer membrane and are therefore known as outer membrane vesicles or OMVs (Jan 2017; Caruana and Walper 2020). Peptidoglucan is a structural component in the cell wall of most bacteria. In gram-negative bacteria, lipopolysaccharides form an external membrane layer outside the peptidoglucan layer. These features combined with the unicellular nature and lack of a nuclear membrane make bacteria amenable to accepting foreign DNA, via processes such as transformation, transduction, and conjugation (Alberts *et al.* 2019). Eukaryotes have a much more complex cell design with membrane-bound nuclei, different types of membrane-enclosed organelles, cytoskeleton, and many other features compared to bacteria. Still, the exchange of nucleic acids occurs between prokaryotic and eukaryotic cells (Bitto *et al.* 2017).

**Fig. 1.**
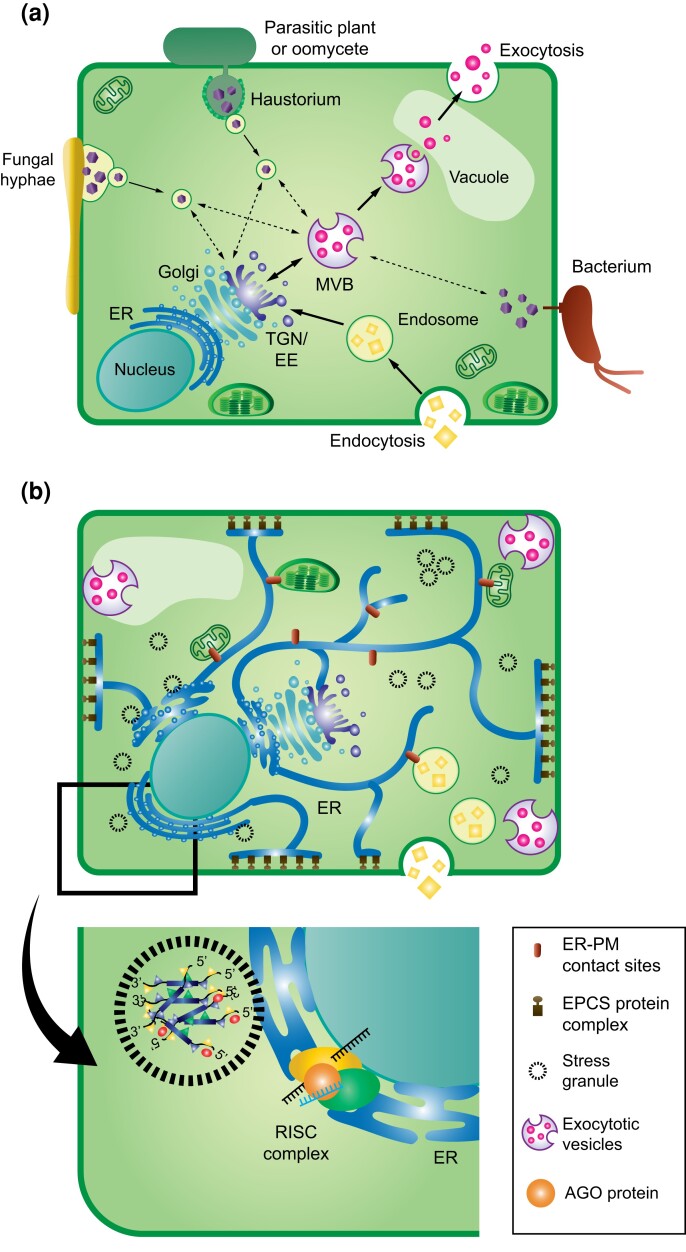
Simplified illustrations of possible DNA and RNA transport routes within and between plant cells and invading organisms. a) In several cases when different organisms have established tight physical relationships with plants, the exchange of various molecules and even organelles in the case of parasitic plants can occur. Several studies have reported on the exploitation of the endo-exocytotic system, which can transport nucleic acids reciprocally across species boundaries (Zhang *et al.* 2019; Xing *et al.* 2021). MVB and TGN/EE are central hubs in vesicle trafficking. Arrows, endo/exocytosis; dotted arrows, suggested translocation of effectors; EE, early endosome; ER, endoplasmic reticulum; MVB, multivesicular body; and TGN, trans-Golgi network. b) Extracellular vesicles and particles comprise heterogenous cargo populations. Vesicles may contain small RNAs bound to AGOs or other types of RBP or alternatively contain DNA (Baldrich *et al.* 2019). How DNA loading is taken place is not fully understood. As an alternative to membrane-enclosed vesicles, RNA could assemble with proteins in membrane-less SGs. The ER forms an essential cellular network interacting through various proteins with the cytoskeleton organization. Membrane-less SGs are suggested to be a faster way of communication than vesicles and be driven by ER (van Leeuwen and Rabouille 2019). PM, plasma membrane; EPCS, ER-PM contact site; RISC, RNA-induced silencing complex; and argonaute, AGO. Illustrations by Cajsa Lithell.

The endomembrane system is a fundamental cellular organization composed of 2 main routes, 1 for outgoing traffic (exocytosis) and the other for incoming traffic (endocytosis) (Fig. 1a). It is believed that small RNAs connect to the endomembrane system in the endoplasmic reticulum (ER) for further vesicle transport in the cell, potentially leading to translocation to other species (Bonifacino and Glick 2004; Kim *et al.* 2014; Lefebvre and Lécuyer 2017; Basyuk *et al.* 2021). Vesicles with DNA- or RNA-containing cargo have contributed to gene transfer between several prokaryotic species (Tashiro *et al.* 2019). Compared to plants, vesicles, their biogenesis, and cargo selection in animal cells are well studied (Maas *et al.* 2017; van Niel *et al.* 2018; Ghanam *et al.* 2022; Dixson *et al.* 2023). Based on today's technologies, vesicles can only become isolated from plants that are exposed to stress (Rutter and Innes 2017; Cai *et al*. 2018; Chalupowicz *et al*. 2023), demonstrating a link between stress and vesicle biogenesis.

There are increasing numbers of reports on parasite or pathogen–host interactions involving different strategies to inject or otherwise transfer molecules for their own benefit and at the same time avoid recognition and triggering of the host immune system (Hua *et al.* 2018; Ruf *et al.* 2022). EVs are proposed to have a key role in these events including reverse small RNA trans-kingdom transfer from the plant host to specifically target RNA-silencing suppressors in the pathogen (Hou *et al.* 2019). Outer membrane vesicle (OMV)–mediated cargo secretion in *Pseudomonas* species has been shown to generate broad-spectrum immune responses to bacterial species and oomycetes in Arabidopsis and tomato (McMillan *et al.* 2021). Based on these data, OMVs seem to mediate previously unknown pathways for plants, ensuring differentiation between different types of microorganisms and initiating selective protection pathways not previously known.

## The ER: a dynamic organellar structure

The ER was discovered for more than 100 years ago, yet we do not have a full understanding of all its functions beyond being the primary site of ribosomes, the mRNA translation machinery, and protein modifications in the cell (Schwarz and Blower 2016). The ER is an interconnected cellular network composed by flat structures (sheets), reticular networks (tubules), and a matrix of the tubules. A number of contact sites between ER and other organelles have been shown or suggested such as with the peroxisomes, chloroplasts, mitochondria, Golgi, and the plasma membrane (Wenzel *et al.* 2022; Wang *et al.* 2023). The ER membrane stack and network links the nuclear envelope with other organelles in the cytoplasm and needs to respond in a proper, timely, and precise way to changes related to alterations in cell cycles, developmental stages, and various stress situations. Such changes require rapid remodeling of the ER structure, including its nuclear positions that impact the cytoskeleton connections (Pain and Kriechbaumer 2020; Janota *et al.* 2022). The secretion pathway starts at the ER with vesicles embraced with coat protein complexes. Plants also form specialized ER-derived vesicles that can exit in a Golgi-independent manner (Chrispeels and Herman 2000; Cheung *et al.* 2022). The ER is considered as a contiguous organelle that is undergoing constant changes, controlled by the actin–myosin complex within the cytoskeleton. This dynamic feature of ER facilitates the flow of heterogenous populations of stress granules (SGs) and vesicles within the cell. How invading organisms exploit this transport system is yet not demonstrated.

## Are minicircles and membrane-less organelle shuttles for intracellular nucleic acid?

The *Beet curly top Iran virus* (BCTIV) is an example of a single-stranded DNA virus that mediates the transfer of sugar beet DNA to other plant hosts (Catoni *et al.* 2018). BCTIV is transmitted by several insect species including whitefly, a notorious virus vector (Fiallo-Olivé *et al.* 2020). Interestingly, as many as 49 plant-like genes have been discovered in its genome (Gilbert and Florian Maumus 2022). Whether those acquired genes explain the wide host adaptation of whitefly resulting in its well-known efficiency as a virus vector remains to be demonstrated.

In the BCTV system, host DNA is hijacked and packed into minicircles that are cotransmitted during the virus infection of the next plant (Catoni *et al.* 2018). In these cases, heterogenous vesicle populations are present making it into a plausible site for sequence exchanges of nucleic acids with different origins.

Many of the membrane-less compartments are large and diverse assemblies of RNA and protein, referred to as ribonucleoprotein (RNP) granules, including the so-called SGs (Fig. 1b). SGs are built of mRNAs, translation factors, RNA-binding proteins (RBPs), and other proteins that coalesce together (Becker and Gitler 2015). SGs are implicated in the regulation of translation, mRNA storage and stabilization, and cell signaling, particularly during abiotic stress conditions in plants and humans (Jang *et al.* 2020; Curdy *et al.* 2023; Kearly *et al.* 2023). SGs are quickly assembled upon stress and then rapidly dispersed when the aggravating state has terminated. SGs interact with processing bodies (P or PB), which are involved in the RNA degradation. Recent data suggest that ER serves as a subcellular site for the SG formation (Lee *et al.* 2020; Child *et al.* 2021; Nicchitta 2022). However, the ubiquitous RNP granules are diverse and proper RNA–RNA interactions are of importance to avoid or limit undesirable aggregates (Ripin and Parker 2022). The ER also plays important roles in the RNA-induced silencing complex (RISC) formation and small RNA processing (Stalder *et al.* 2013; Li *et al.* 2016; Axtell 2017). The possibility that ER could act as a cross-road for RNA or DNA molecules with different species affiliations to be transferred to separate cellular destinations is tempting.

Most information on SGs in plants so far derives from virus infections. One example is the cauliflower mosaic virus. This virus manipulates the host cell and alleviates translational repression by targeting the PBs, which are aggravated under uninfected conditions (Hoffmann *et al.* 2022). Further insights into how viruses manipulate cellular pathways are now coming from multiple sources (Ravindran *et al.* 2016; Thaker *et al.* 2019; Irwin *et al.* 2022). We anticipate that they will pave the way for new understandings of intracellular events, intracellular mechanisms behind cell-to-cell movements, and interorganismal communications.

## Final remarks

The flow of nucleic acids between plants and organisms in immediate close proximity seems to occur with different intensities and host acceptance levels. First, during extreme challenges, new traits and even organelles have been accepted by taxa in Viridiplantae, resulting in fitness advantages that eventually lead to survival of individual species (Martin et al. 2015; Clark and Donoghue 2018; Cheng *et al.* 2019; Su *et al.* 2021). An interesting rather recent discovery is the promiscuous allele in the legume *Lotus burtii* allowing nodulation by 5 Rhizobium species classified into different taxonomic genera (Zarrabian *et al.* 2022).

Reports from a multitude of eukaryotic organisms including plants point to the fact that nucleic acids are transported within and between cells and organisms as a rapid response to changed environments. Plants do acquire nucleic acid from invading organisms, but the expression can be suppressed by host-induced gene silencing (Nunes and Dean 2012). There are many examples of pathogens that hijack the host cell machinery or following entry can exploit or manipulate host cell organelles for their own benefit (Figueroa *et al.* 2021; Kellermann *et al.* 2021). Pathogen attacks and impact of abiotic factors such as temperature changes require rapid adaptation of a plant cell. In the latter case, SGs are reported to quickly impact translation or transcription levels of selected sequences (Maruri-López *et al.* 2021; Nicchitta 2022).

The picture is complex and many questions remain to be answered about sequence selectivity, intracellular transmission, and modification processes. The frequency of transient influences in somatic cells is most likely much greater than an impact on gametes. It is unclear if, when, and how the latter step is taken. To advance our understanding of these dynamic events, we foresee progress based on new microscopic advances (Colin *et al.* 2022) and single molecule techniques in combination with new biological tools in order to monitor small quantities of fast-moving molecules, sequence modifications, and organelle engagements.
